# Bochdalek Hernia Revealed by Incarceration of the Splenic Flexure: A Rare Case in an Adult

**DOI:** 10.1155/cris/9974415

**Published:** 2026-02-26

**Authors:** Mohammad Al jazaerly, Tareq Wehba, Ali Hmidoush, Jamal Ataya, Yassine Younesse

**Affiliations:** ^1^ General Surgery, Damascus Hospital, Damascus, Syria, damascushospital.org.sy; ^2^ Faculty of Medicine, Tishreen University, Latakia, Syria, tishreen.edu.sy; ^3^ Faculty of Medicine, University of Aleppo, Aleppo, Syria, alepuniv.edu.sy

**Keywords:** Bochdalek hernia, case report, splenic flexure incarceration, Syria

## Abstract

**Introduction:**

Bochdalek hernia (BH), a congenital anomaly that typically manifests in infancy, occasionally presents in adults and can lead to serious complications. “Splenic flexure incarceration” within a BH is a rare but clinically significant event, in which the colonic segment near the spleen becomes entrapped, leading to potential acute complications.

**Case Presentation:**

A 51‐year‐old male with no significant medical history presented with acute abdominal pain, nausea, and an absence of bowel movements. Diagnostic imaging revealed a left‐sided BH with splenic flexure and incarcerated omentum, causing marked colonic distension. Emergency surgery was performed, successfully releasing the adhesions and restoring colonic integrity. The patient’s postoperative course was unremarkable, and he was discharged in good health.

**Conclusion:**

This case highlights the rarity of adult BH with splenic flexure incarceration, and the need for swift surgical intervention to prevent life‐threatening sequelae.

## 1. Introduction

Bochdalek hernia (BH) is a common type of congenital diaphragmatic hernia (CDH) that results from a defect in the posterolateral diaphragm during fetal development, often occurring on the left side in 75%–90% of cases [1, 2]. In adults, BHs are often incidentally detected during imaging for other reasons, making diagnosis challenging due to nonspecific or asymptomatic symptoms [2, 3]. Although rare, symptomatic BH can cause severe complications, such as volvulus, gastric perforation, and splenic rupture [1, 4, 5]. We report the case of a 51‐year‐old man with bowel obstruction due to an incarcerated splenic flexure in a left‐sided adult‐onset BH.

## 2. Case Presentation

**Figure 1:**
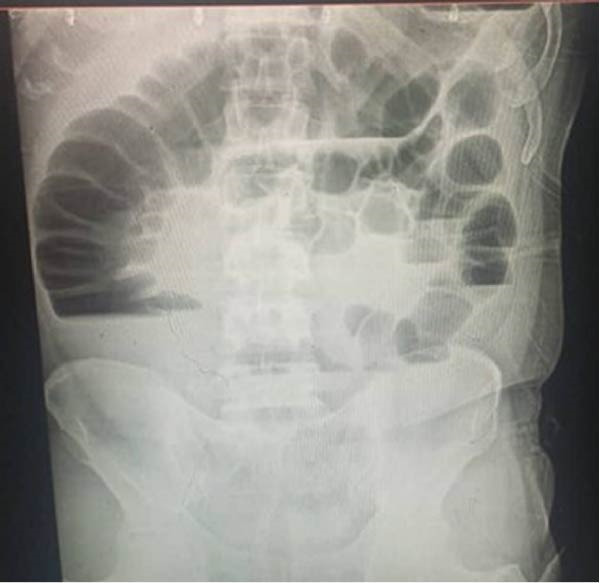
Plain X‐ray image shows multiple air‐fluid levels with significant colonic distension and an absence of gas in the rectum, suggesting colonic obstruction.

**Figure 2:**
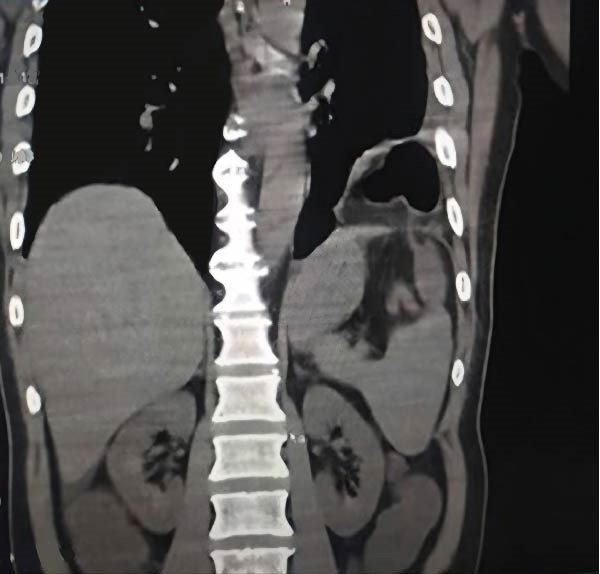
A coronal CT image showing the incarceration of the splenic flexure with omentum from the abdomen within a diaphragmatic hernia.

**Figure 3:**
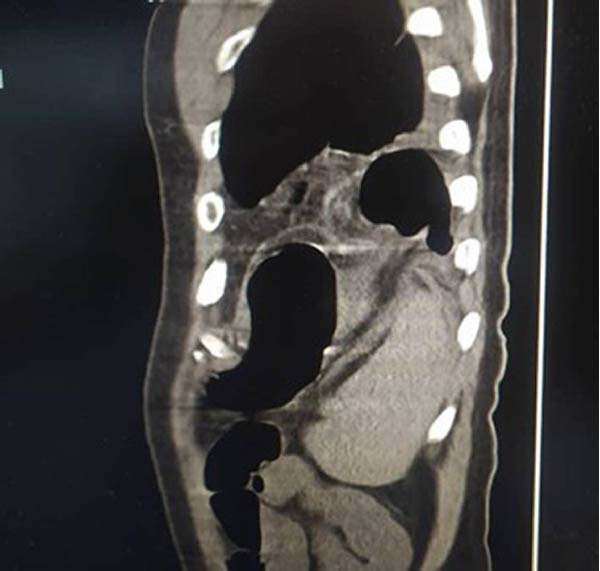
A sagittal CT image showing the incarceration of the splenic flexure with omentum from the abdomen within a diaphragmatic hernia.

A 51‐year‐old nonsmoker man presented to the emergency department with a 4‐day history of generalized abdominal pain, primarily affecting the epigastric region and left upper quadrant. The patient also experienced nausea and notable absence of bowel movements and gas. The patient had no prior medical history, and the family history revealed the mother’s migraines. Upon examination, the abdomen appeared tender and distended. The blood pressure was 90/60 mmHg, and pulse rate was 115 beats/min. Given the severity of symptoms, urgent radiological assessment is necessary. Echography showed free fluid in the abdomen and pelvis, while radiography indicated colonic obstruction with multiple air‐fluid levels and absent gas in the rectum (Figure 1). A computed tomography (CT) scan revealed a small left pleural effusion, a 2.7 cm left diaphragmatic hernia containing the splenic flexure and omentum, marked distension of the transverse and ascending colon (~9 cm), collapse of the descending and sigmoid colon, and free pelvic fluid (Figures 2 and 3). Laboratory results showed leukocytosis (15,200 cells/µL), normal electrolyte levels with sodium 131 mEq/L, potassium 3.3 mEq/L, chloride 92 mEq/L, bicarbonate Hco_3_^-^ 24 mEq/L, urea 38.4 mg/dL, creatinine 0.66 mg/dL, alanine aminotransferase (ALT) 14 U\L, aspartate aminotransferase (AST) 16 U\L, alkaline phosphatase (ALP) 50 U\L, gamma‐glutamyl transferase (GGT) 20 U\L, bilirubin 0.3 mg\dL, albumin 4 g\dL, and total protein 7 g\dL. Creatine phosphokinase was slightly elevated at 145 U/L, and the C‐reactive protein was 39.3 mg/dL, suggesting significant inflammation.

Under general anesthesia, midline laparotomy was performed. Upon entering the abdominal cavity, severe distension of the transverse and ascending colon was noted, with the splenic flexure incarcerated within a 2.7 cm left diaphragmatic defect. The herniated splenic flexure and omentum were carefully released into the abdominal cavity after lysing dense adhesions between the colon and diaphragmatic rim. Colon viability was confirmed by restoring its color, peristalsis, and pulsatile mesenteric blood flow (Supporting Information).

Thorough exploration of the abdominal cavity revealed no associated congenital anomalies, such as intestinal malrotation, mesenterium commune, situs inversus, or hepatic malformations. The diaphragmatic defect was repaired primarily using interrupted horizontal mattress sutures (0 Ethibond) to approximate the muscular edges and ensure tension‐free closure. Given the defect size (2.7 cm) and risk of recurrence, a synthetic polypropylene mesh (15 × 10 cm) was placed in an underlay fashion and secured with nonabsorbable sutures to reinforce the repair. The abdominal cavity was irrigated, and a pelvic drain was placed to monitor postoperative fluid accumulation. The fascia and skin were closed in these layers.

## 3. Postoperative Course

The patient’s recovery was closely monitored and progressed favorably. He was managed with intravenous fluids and analgesics. A liquid diet was introduced on postoperative day 1, which was successfully advanced to a soft diet on day 2 as tolerated. The patient passed flatus on postoperative day 2. The pelvic drain was removed on postoperative day 3 after the output was noted to be minimal and serosanguinous. The patient remained afebrile, and his leukocytosis gradually normalized. The patient was discharged on postoperative day 5 in a stable condition, with scheduled follow‐up appointments for clinical evaluation and planned imaging to assess the integrity of the diaphragmatic repair.

## 4. Discussion

Congenital diaphragmatic hernia (CDH), a developmental defect permitting abdominal organ herniation into the thorax, encompasses BH and was first described in 1754 and later characterized by Bochdalek in 1848. This type of CDH is more common on the left side (85%), as observed in the present case [1, 3].

While BH exhibits a 3:1 male predominance [6], symptomatic adult presentations—like our 51‐year‐old male with acute bowel obstruction—are exceedingly rare [1], as seen in our 51‐year‐old male patient. The size of the diaphragmatic defects can vary significantly [4]. BH often involves herniation of the abdominal organs, including the omentum (92%), splenic flexure of the colon (58%), stomach (25%), and spleen, and is commonly found on the left side [1]. Our patient had a left‐sided hernia (2.7 cm) with herniated splenic flexure and no associated anomalies (e.g., ectopic kidney or other organ involvement in the diaphragmatic defect) like kumar case that reported [4].

BH may be asymptomatic or present with other symptoms [1]. In a review by Brown et al. [7], only 14% of 173 cases presented with symptoms. Symptomatic patient symptom type and severity depend on the presence, severity, and acuteness of herniation. Children usually present with respiratory distress, whereas adults experience more chronic symptoms such as dyspnea and chest pain. Abdominal pain is common in adults with BH, while intestinal obstruction or signs of perforation can occur in both [4, 6, 8]. Our patient presented with abdominal pain, obstipation, and vomiting.

Symptoms can also be categorized as either intermittent or acute, depending on the extent of herniation. Acute presentations often arise from complications such as incarceration, obstruction, gastric volvulus, splenic rupture, or strangulation of the herniated viscera, requiring prompt intervention [4, 8]. In our case, bowel obstruction and splenic flexure incarceration were observed; however, no signs of ischemia or volvulus were observed.

Diagnosis of BH in adults remains challenging because of its variable presentation. Although prenatal ultrasound detects most cases by 24 weeks’ gestation [9], many remain undetected until [5]. Many BH cases are asymptomatic and discovered incidentally during routine imaging, such as chest radiography or CT scans [5, 8]. This underscores the importance of considering BH in differential diagnoses [5].

In symptomatic cases, diagnosis remains elusive. Nonspecific symptoms such as abdominal pain or chest tightness can lead to misdiagnosis [5]. Various imaging modalities are available, including chest and abdominal radiography, fluoroscopy, barium examination, ultrasound, CT, and MRI. Contrast‐enhanced CT has emerged as the gold standard for definitive diagnosis [1], provides superior visualization of thoracic contents, allows for clear identification of diaphragmatic defects and herniated abdominal contents [3], and offers detailed information about defect size, with a sensitivity of 78% for left‐sided hernias [1]. However, this does not completely rule out the possibility of BH [5]. Surgical repair of BH involves repositioning the herniated abdominal contents and repairing the diaphragmatic defects. Since 1995, minimally invasive techniques have gained popularity in surgeries for left‐sided hernias, which are traditionally performed through an abdominal approach, and right‐sided hernias, which require a thoracic approach [8]. However, while current practices consider broader factors and minimally invasive techniques are increasingly preferred for minor defects in asymptomatic cases [2, 5], an exploratory laparotomy was necessary in our case to liberate the splenic flexure, assess its viability, and rule out necrosis. As a matter of fact, both serosal tear of the colonic wall [10] and ischemia of the splenic flexure requiring extended right hemicolectomy [11] have been reported. Since conservative management for BH is not recommended, in complex cases, if not feasible by laparoscopy, conversion to open surgery may be necessary to ensure the best possible outcomes [5].

## 5. Conclusion

This case underscores the importance of identifying and managing a rare BH in adults because overlooking it can lead to serious complications. Resolution of splenic flexure incarceration highlights the need for prompt surgical intervention in such cases.

**Nomenclature**
BH: Bochdalek hernia
CDH: Congenital diaphragmatic hernia.

## Author Contributions

Tareq Wehba and Ali Hmidoush wrote the manuscript and reviewed relevant literature. Mohammad Al jazaerly was the main surgeon responsible for the following aspects of patient care. Jamal Ataya revised and prepared the manuscript. Jamal Ataya and Yassine Younesse supervised this case.

## Funding

No funding was received for this research.

## Disclosure

All the authors have read and approved the final manuscript.

## Consent

Written informed consent was obtained from the patient for publication of this case report and accompanying images. A copy of the written consent form is available for review by the Editor‐in‐Chief of this journal upon request.

## Conflicts of Interest

The authors declare no conflicts of interest.

## Supporting Information

Additional supporting information can be found online in the Supporting Information section.

## Supporting information


**Supporting Information** A surgical video clip demonstrating the release of the incarcerated splenic flexure from the diaphragmatic defect is available as supporting information.

## Data Availability

Data sharing is not applicable to this article, as no new data were created or analyzed in this study.
